# RRM1 Expression as a Prognostic Biomarker for Unresectable or Recurrent Biliary Tract Cancer Treated with Gemcitabine plus Cisplatin

**DOI:** 10.3390/jcm10204652

**Published:** 2021-10-11

**Authors:** Jung Won Chun, Boyoung Lee, Weon Seo Park, Nayoung Han, Eun Kyung Hong, Eun Young Park, Sung Sik Han, Sang-Jae Park, Tae Hyun Kim, Woo Jin Lee, Sang Myung Woo

**Affiliations:** 1 Center for Liver and Pancreatobiliary Cancer, National Cancer Center, Goyang-Si 10408, Gyeonggi-Do, Korea; deli4927@snu.ac.kr (J.W.C.); bylee11050724@gmail.com (B.L.); hanny@ncc.re.kr (N.H.); hongek@ncc.re.kr (E.K.H.); sshan@ncc.re.kr (S.S.H.); spark@ncc.re.kr (S.-J.P.); k2onco@ncc.re.kr (T.H.K.); lwj@ncc.re.kr (W.J.L.); 2 Department of Pathology, National Cancer Center, Goyang-Si 10408, Gyeonggi-Do, Korea; thymus@ncc.re.kr; 3 Biometrics Research Branch, Research Institute, National Cancer Center, Goyang-Si 10408, Gyeonggi-Do, Korea; 13140@ncc.re.kr

**Keywords:** biliary tract neoplasms, cisplatin, gemcitabine, ribonucleotide reductases

## Abstract

The combination of gemcitabine plus cisplatin (GP) is regarded as a first-line treatment for patients with unresectable or recurrent biliary tract cancer (BTC). Several proteins including human equilibrative nucleoside transporter-1 (hENT1), deoxycytidine kinase (DCK), cytidine deaminase (CDA), and ribonucleotide reductase subunit 1 (RRM1) are known to be involved in gemcitabine uptake and metabolism. This study was aimed to identify the predictive and prognostic values of these biomarkers in patients who treated with GP for advanced BTC. Tumor samples were obtained from 34 patients with unresectable or recurrent BTC who were treated with GP between August 2015 and February 2018. Intratumoral expression of hENT1, DCK, CDA and RRM1 was determined by immunohistochemistry and analyzed for association with chemotherapy response, progression-free survival (PFS) and overall survival (OS). Median OS was significantly longer in the RRM1-negative group than in the RRM1-positive (9.9 months vs. 5.9 months, *p* = 0.037). Multivariate adjustment analyses also demonstrated RRM1 expression as an independent prognostic factor for OS in patients treated with GP chemotherapy. Increased intratumoral expression of RRM1 on immunohistochemical staining may be a biomarker predicting poor survival in patients with GP chemotherapy for advanced BTC. Large-scale well-predefined prospective research is needed to validate the utility of biomarkers in clinical practice.

## 1. Introduction

Biliary tract cancers (BTCs) are uncommon malignant neoplasms of the gastrointestinal tract, which consist of a group of heterogeneous tumors including gallbladder (GB) cancer, cholangiocarcinoma (CCA) of the extra- and intra-hepatic bile ducts, and the ampulla of Vater [1,2]. Most patients with BTC have advanced, unresectable cancer at an initial diagnosis, and have poor survival outcomes with a median overall survival (OS) of less than 1 year [2]. In patients with unresectable BTC, palliative systemic chemotherapy is the mainstay treatment strategy [3]. Although several studies have reported that gemcitabine and gemcitabine-based chemotherapy regimens are effective in patients with advanced BTC, the combination of gemcitabine and cisplatin (GP) is now accepted as the standard first-line chemotherapy in these patients [4,5,6,7].

Predicting patient response to chemotherapeutic agents would be useful in selecting appropriate patients for chemotherapy and in predicting survival outcomes. Several transporters, including human equilibrative nucleoside transporter-1 (hENT1) and human concentrative nucleoside transporters (hCNT), and several enzymes, such as deoxycytidine kinase (DCK), deoxycytidine deaminase (CDA), and ribonucleotide reductase M1 (RRM1), were found to be involved in gemcitabine uptake and metabolism [8,9]. Further, these proteins have been suggested as predictors for the efficacy of gemcitabine treatment and prognostic biomarkers in several cancer cell lines and specimens [10,11,12,13].

In BTCs, expression levels of hENT1 and RRM1 have also been associated with gemcitabine sensitivity [14,15,16]. A recent systematic review and meta-analysis, which evaluated the prognostic value of 26 immunohistochemical biomarkers in 1348 patients with BTC, suggested that hENT1 may be a promising biomarker for patient response to gemcitabine-based chemotherapy [17]. The study mainly discussed the results of hENT1, RRM1, and Excision Repair Cross-Complementation 1, which were frequently evaluated biomarkers. However, the prognostic value of the two other biomarkers except hENT1 showed unclear results, and the remaining 23 biomarkers including CDA and DCK were not discussed in detail due to the limited number of studies.

Our group has previously reported the predictive and prognostic values of CDA and DCK in BTC. Although the polymorphism of CDA was associated with tumor response in unresectable, metastatic BTC patients treated with GP chemotherapy, immunohistochemical evaluation was not performed [18]. Further, DCK positivity was significantly associated with longer recurrence-free survival in resected BTC with postoperative gemcitabine chemotherapy [19]. However, most patients with BTC are not indicated for curative intent surgery at initial diagnosis, and GP chemotherapy is the most effective regimen for advanced BTC. Therefore, in this study, we aimed to evaluate the predictive and prognostic value of key molecules (hENT1, DCK, CDA, and RRM1) in gemcitabine metabolism by analyzing the intratumoral expression in tumor samples and clarify which biomarker is the most reliable for patients treated with GP chemotherapy for unresectable, metastatic and recurrent BTC.

## 2. Materials and Methods

### 2.1. Patients and Data Collection

From August 2015 through February 2018, consecutive patients with advanced unresectable or recurrent BTC who received GP chemotherapy were prospectively enrolled in this study. BTC is comprised of GB cancer, intrahepatic and extrahepatic CCA. The study protocol was approved by the Institutional Review Board of the National Cancer Center, Korea (IRB No. NCC2015-0113) and was conducted in accordance with the provisions of the Declaration of Helsinki. All participants provided written informed consent before recruitment.

The combination chemotherapy regimen consisted of cisplatin 25 mg/m^2^ and gemcitabine 1000 mg/m^2^ administered intravenously on days 1 and 8 every 3 weeks. Patients were included if they had at Eastern Cooperative Oncology Group (ECOG) performance status (PS) of 0 to 2 and adequate organ function, including an absolute neutrophil count ≥ 1500/mm^3^; a platelet count ≥ 100,000/mm^3^; serum creatinine ≤ 1.5 X the upper limit of normal; total bilirubin ≤ 1.5 mg/dL; and either AST or ALT 2× the upper limit of normal at baseline. Patients were excluded if they had received prior anticancer therapy for the current malignancy or if their tumor type was other than adenocarcinoma.

Tumors were evaluated by the Response Evaluation Criteria in Solid Tumors (RECIST) every three cycles during chemotherapy or earlier if clinically indicated using appropriate tumor imaging, such as computed tomography (CT) and magnetic resonance imaging (MRI). The best overall response for each patient was recorded. The decision to discontinue treatment was based on disease progression, the patient’s choice, the clinician’s judgment, or unacceptable treatment-related adverse events (AEs). Progression-free survival (PFS) was defined as the time from the date of subject enrollment until tumor progression on imaging or death, and OS was defined as the time from subject enrollment to the date of death. All AEs were graded according to the National Cancer Institute’s Common Toxicity Criteria (version 4.0).

### 2.2. Preparation and Immunostaining of Specimens

In patients with advanced unresectable BTC, tumor biopsy specimens for pathologic diagnosis were obtained by experienced radiologists. Needle biopsies were performed using a freehand technique under real-time ultrasound guidance, targeting primary or metastatic tumor tissue. Usually, two biopsy samples were obtained from each tumor mass using an 18-gauge biopsy device under ultrasound guidance. In recurrent cases after surgery, primary resection specimens were used for the evaluation of marker expression. All specimens were independently examined by two pathologists (W.S.P. and E.K.H). Tumors were classified as well-differentiated, moderately differentiated, or poorly differentiated adenocarcinomas, based on predominant pathological grading. Hematoxylin and eosin-stained slides containing specimens from each BTC sample were reviewed, and a representative tumor region and the corresponding formalin-fixed paraffin-embedded tissue block were selected for immunohistochemistry (IHC). One to three paraffin-embedded blocks (median, two blocks) of each specimen were evaluated by IHC. Three serial 3-μm sections were cut and prepared from each block: one for hematoxylin-eosin staining; one for immunostaining with the indicated primary antibodies against proteins associated with gemcitabine transport and metabolism; and one as a negative control. Primary antibodies included rabbit polyclonal antibodies to human CDA (catalog number: 48-962, ProSci, Inc., Poway, CA, USA), human DCK (catalog number: LS-B1825, LifeSpan Bioscience, Inc., Seattle, WA, USA), human hENT1 (catalog number: 11337-1-AP, ProteinTech Group, Inc., Chicago, IL, USA), and human RRM1 (catalog number: 10526-1-AP, ProteinTech Group, Inc.) [16].

Immunoreactivities of proteins were assessed by two experienced pancreaticobiliary pathologists. Protein expression of tumor cells was scored by immunostaining intensities as follows: grade 0, not stained; grade 1, faint positive; grade 2, weak to moderate positive; grade 3, strong positive. The staining intensity of tumor was determined by the most intense staining tumor cell. Discrepancies between the two pathologists were settled by the use of a multiheaded microscope to arrive at a final consensus.

### 2.3. Statistical Analysis

The primary outcomes were OS and PFS. Median follow-up was calculated using the reverse Kaplan–Meier method [20]. Survival was estimated using the Kaplan–Meier method and compared by log-rank tests. The associations of clinicopathological factors with OS were analyzed using Cox proportional-hazards regression models. Factors with *p*-values < 0.1 on univariate analysis were included in the multivariate regression model, along with the immunoreactivity of each intratumoral protein marker. The multivariate Cox model was selected using backward stepwise selection to eliminate non-significant variables at *p* < 0.1. Hazard ratios (HRs) were calculated, along with their associated 95% confidence intervals (CIs). Statistical analyses were performed using SAS statistical software (version 9.3) and R (version 3.3.1) and all reported p-values are two-sided.

## 3. Results

### 3.1. Patient Characteristics

A total of 34 patients with advanced BTC were enrolled during the study period (Table 1). Sixteen (47%) of these patients were diagnosed with GB cancer, followed by 10 (29%) with intrahepatic and eight (24%) with extrahepatic CCA. Among them, 10 patients (extrahepatic CCA: 7; GB cancer: 2, intrahepatic CCA: 1) were recurrent cases, and the median time to recurrence after surgery was 9.4 months (range, 1.4–20.2 months). Assessments of the degree of differentiation showed that two (6%) tumors were well-differentiated, 16 (47%) were moderately differentiated, and 13 (38%) were poorly differentiated adenocarcinomas. Twenty-four patients (71%) had metastatic disease, with the most common metastatic sites being distant lymph nodes in 20 patients and the liver in 16 patients.

### 3.2. Immunohistochemistry Staining of Biomarker Expression

**Figure 1:**
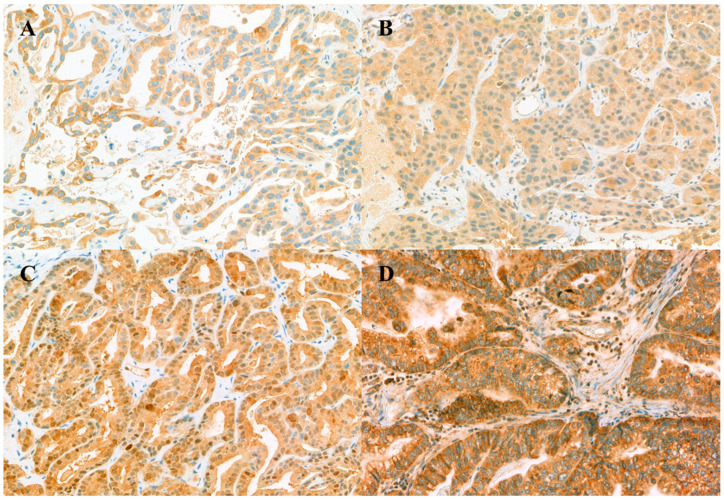
Immunohistochemical analysis of the intratumoral expression of proteins associated with gemcitabine transport and metabolism in patients with biliary tract cancers. (**A**) hENT1 in gallbladder (GB) cancer, (**B**) DCK in intrahepatic cholangiocarcinoma, (**C**) CDA in GB cancer, (**D**) RRM1 in GB cancer.

Representative examples of positive immunohistochemical labeling profiles are shown in Figure 1. Cellular staining was localized to the cytoplasm for hENT1, DCK, and RRM1, the nucleus and cytoplasm for CDA. Immunohistochemical labeling was defined as positive when hENT1, DCK, and CDA were 1+ or higher, and RRM1 was 2+ or higher. Of the 34 patients, hENT1-positive was in 25 (73.5%), DCK-positive in 11 (32.4%), CDA-positive in 12 (36.4%), and RRM1-positive in 10 (30.3%). Negative examples are also presented in Figure S1. There were no significant differences in clinicopathological characteristics based on the positivity of each biomarker, except that GB cancers were frequently observed in hENT1-positive and metastatic disease was common in DCK-positive (Table S1).

### 3.3. Treatment Outcomes and Intratumoral Biomarker Expression

During the median follow-up of 34.2 months (95% CI, 5.2–63.2 months), 31 (91%) of the 34 patients experienced disease progression and died. Patients received a median of 5 cycles (range, 1–18 cycles) of chemotherapy. Three patients (8.8%) showed partial responses to GP treatment and the disease control rate (DCR) was 58.8%. Median OS and PFS were 7.5 months (95% CI, 5.4–9.6 months) and 4.4 months (95% CI, 3.4–5.4 months), respectively.

The association between chemotherapy effectiveness and biomarker expression was evaluated in Table 2. Expression of hENT1, DCK, CDA, and RRM1 was not significantly associated with response to GP chemotherapy.

**Figure 2:**
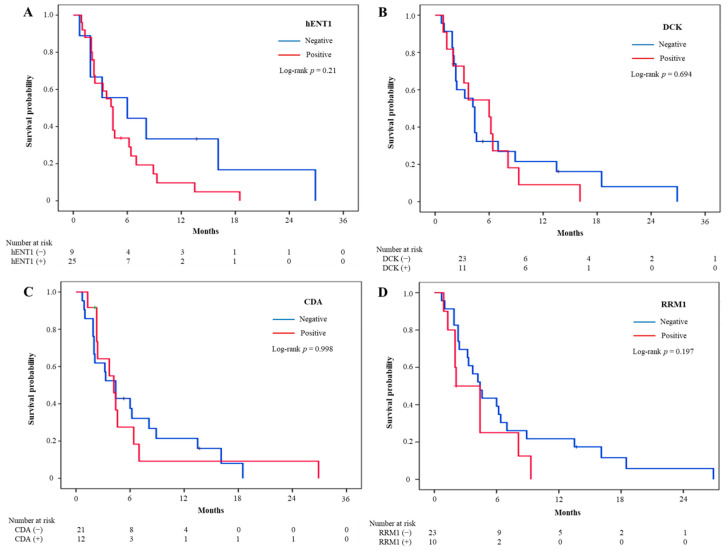
Kaplan–Meier analysis of the relationships of intratumoral expression of biomarkers with progression-free survival in patients with biliary tract cancers. (**A**) hENT1, (**B**) DCK, (**C**) CDA, (**D**) RRM1. hENT1, human equilibrative nucleoside transporter 1; DCK, deoxycytidine kinase; CDA, Cytidine deaminase; RRM1, ribonucleotide reductase subunit 1.

**Figure 3:**
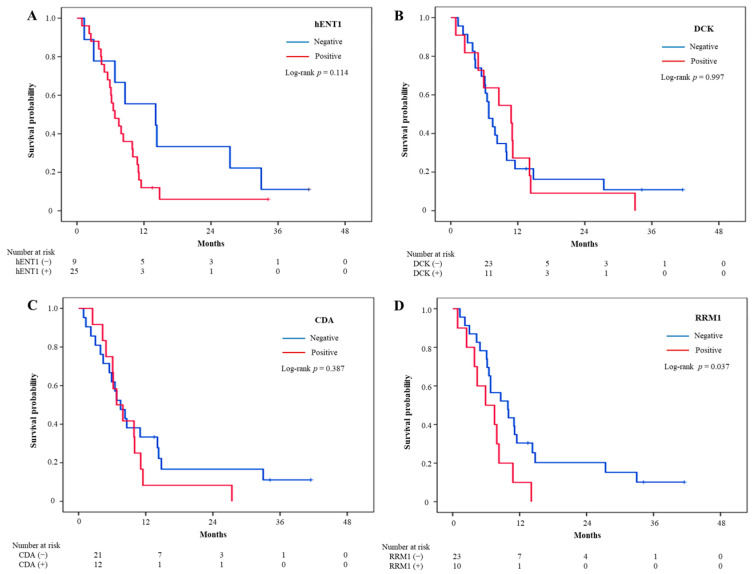
Kaplan–Meier analysis of the relationships of intratumoral expression of biomarkers with overall survival in patients with biliary tract cancers. (**A**) hENT1, (**B**) DCK, (**C**) CDA, (**D**) RRM1. hENT1, human equilibrative nucleoside transporter 1; DCK, deoxycytidine kinase; CDA, Cytidine deaminase; RRM1, ribonucleotide reductase subunit 1.

Survival analysis was performed according to each biomarker. There was no significant difference in survival between positive and negative in hENT1, DCK, and CDA. Median PFS was longer in RRM1-negative (4.4 months; 95% CI, 3.0–5.8 months) than in RRM1-positive (2.1 months; 95% CI, 0–4.3 months) patients (Figure 2). However, this trend did not reach statistical significance. On the other hand, median OS was significantly longer in RRM1-negative than in RRM1-positive (9.9 months vs. 5.9 months, *p* = 0.037) (Figure 3). 

The prognostic effect of intratumoral protein markers on survival in patients with BTC was assessed by Cox proportional hazards modeling based on the expression of CDA, hENT1, DCK, and RRM1. Univariate analyses showed that the number of metastatic sites and lung metastasis were significantly associated with PFS (Table 1). Further, the number of metastatic sites and peritoneal seeding, and RRM1 expression were significantly associated with OS (Table 1 and Table 2). Multivariate analyses adjusted by variables with *p* < 0.1 on univariate analyses were performed according to each biomarker. Potential confounding factors (number of metastatic sites and lung metastasis or peritoneal seeding) could not be included simultaneously in a multivariate analysis, so the number of metastatic sites was separately added to the multivariate analysis for each biomarker. Multivariate-adjusted hazard ratios of each biomarker for overall survival were presented in Table 3 and Table S3. RRM1 positivity was significantly associated with poorer OS, whereas there was a nonstatistically significant association with PFS (Table S2). Other biomarkers were not associated with survival outcomes.

## 4. Discussion

The prognosis of patients with BTC remains poor, with GP chemotherapy being the main treatment option for patients with unresectable BTC [3]. No definitive prognostic biomarkers for survival have been available in the clinical setting for patients with advanced BTC [21]. Because of its wide applicability and cost-effectiveness, IHC is frequently used to identify biomarkers associated with survival [17]. The present study evaluated the prognostic ability of intratumoral expression of hENT1, DCK, CDA and RRM1 to assess survival in patients with advanced BTC treated with GP chemotherapy. OS was significantly longer in patients with RRM1-negative than RRM1-positive tumors, and the result demonstrated that the expression of RRM1 was an independent predictor for survival in patients treated with GP for metastatic or recurrent BTC. 

The prognostic or predictive activity of RPM1 expression has been evaluated preclinically and clinically in various types of cancer [22]. For example, several retrospective studies have evaluated the association between RRM1 expression and survival outcomes in patients with BTC. Although two studies found that low RRM1 expression was significantly associated with prolonged OS [23,24], two other studies found no significant differences between patients with high and low RRM1 expression [15,25]. The present study found that RRM1-negative was significantly associated with prolonged OS. Although the small number of the study population was limited in showing the survival difference with sufficient power, there were no significant differences in baseline characteristics between RRM1-positive and negative groups. The prognostic significance of RRM1 was also made both in univariate and multivariate analysis. RRM1 is a crucial enzyme catalyzing the conversion of ribonucleoside diphosphates to deoxyribonucleoside diphosphates, which are utilized for DNA synthesis and repair [22,26]. Gemcitabine, an analogue of deoxycytidine, and its metabolite inhibit RRM1 activity by binding to one of its subunits [22]. In this regard, RRM1 abundance might reduce the cytotoxic effect of gemcitabine on DNA synthesis and affect poor survival outcomes in advanced BTC patients. Previous studies reported RRM1 as a potential predictive biomarker of gemcitabine in CCA, the expression of RRM1 in this study, however, did not show an association with chemotherapy response, limiting its use as a predictive biomarker of GP chemotherapy efficacy [24,27].

The most frequently studied IHC biomarkers in patients with BTC are hENT1, RRM1 and ERCC1 [17]. High hENT1 expression has been shown to be a biomarker of prolonged patient survival in patients with unresectable BTC who were treated with gemcitabine monotherapy or a gemcitabine-based regimen other than GP [14,15,28]. The current study, however, found that hENT1 was not associated with OS and PFS. DCK expression, which is significantly associated with PFS in our previous prospective study including patients with resected BTC, was not associated with survival outcomes in this study [19]. Gemcitabine is transported into the cell through the hENT1 and is activated by DCK or catabolized by CDA. Phosphorylated gemcitabine, the active form of gemcitabine, is incorporated into DNA to exhibit a cytotoxic effect and further inhibits DNA synthesis by suppressing RRM1. In considering these mechanisms, we assumed that the direct cytotoxic effect of gemcitabine appears after the phosphorylation, and therefore the expression of RRM1 related to activated gemcitabine would reflect survival outcomes in patients treated with GP chemotherapy compared to other biomarkers. 

The present study had several limitations. First, the sample size was small, which is the most important consideration in interpreting the results of this study. BTC is a rare disease, and the rarity of this disease has been an unresolved constraint on patient collection. As BTCs are a highly heterogeneous group of GB cancer, intrahepatic and extrahepatic CCA, it also needs to be counted. In our study, a relatively high proportion of patients diagnosed with GB cancer (47%) were enrolled. Therefore, a further multicenter design study that can adequately include sufficient numbers to ensure statistical representation is warranted. To the best of our knowledge, few studies have concurrently analyzed three or more biomarkers in sufficient samples of unresectable BTCs. Concurrent analysis of biomarkers could be a benefit for interpreting results in consideration of interactions between each biomarker. Furthermore, most studies evaluating the prognostic efficacy of IHC markers were retrospective in design; for example, a recent systemic review and meta-analysis of 26 articles included only two phase-II studies [17,19,29]. Therefore, the prospective nature of the present study would be overcome some of the limitations of retrospective studies, such as collecting tissue samples and clinical data. Finally, the absence of an optimal IHC protocol is a significant hurdle in investigating and comparing the effectiveness of biomarker expressions. Previous studies assessed the expression level of the biomarker at the cut-off for positivity with the percentage of staining tumor cells over 50% or H-score of 6 points, which was calculated as the product of the staining intensity and the percentage of positive cells [17]. On the other hand, our immunostaining intensity and positivity were determined by the most strongly stained tumor cell. Consequently, positivity could be overestimated and contribute to the inconsistency with previously reported results. However, our approach to the staining intensity would have the advantage of being simplified and can be evaluated quickly. Large-scale well-predefined prospective research is needed to develop and standardize IHC protocol and validate the utility of biomarkers in clinical practice. 

## 5. Conclusions

In conclusion, the present study found that RRM1 expression was significantly associated with OS in patients with unresectable BTC who were treated with GP chemotherapy. RRM1 expression may therefore be a potential prognostic biomarker for advanced BTC patients. A large-scale, prospective validation of this biomarker is warranted.

## Figures and Tables

**Table 1:** Clinicopathological characteristics and univariate analysis of survival outcomes.

Variables	N (%)	Univariate (Overall Survival)			Univariate (Progression-Free Survival)		
		*n*	HR (95% CI)	*p*	*n*	HR (95% CI)	*p*
Age, median (range)	65.5 (45–81)	31	1.00 (0.96–1.05)	0.859	31	1.01 (0.96–1.06)	0.653
Sex							
Male	20 (58.8)	19	1		20	1	
Female	14 (41.2)	12	0.78 (0.38–1.61)	0.498	11	0.62 (0.29–1.33)	0.215
Primary tumor site							
Extrahepatic	8 (23.5)	8	1	0.566	7	1	0.632
Gallbladder	16 (47.1)	13	0.78 (0.32–1.89)	0.574	15	1.10 (0.42–2.85)	0.852
Intrahepatic	10 (29.4)	10	1.21 (0.47–3.11)	0.690	19	1.56 (0.55–1.46)	0.404
Pathological differentiation							
Well	2 (6.5)	2	1	0.441	2	1	0.193
Moderately	16 (51.6)	14	0.74 (0.16–3.34)	0.691	14	0.63 (0.14–2.94)	0.556
Poorly	13 (41.9)	12	0.46 (0.10–2.13)	0.317	12	0.32 (0.06–1.57)	0.159
Stage							
Metastatic disease	24 (70.6)	21	1		22	1	
Recurrent disease	10 (29.4)	10	0.87 (0.40–1.85)	0.708	9	0.62 (0.27–1.41)	0.252
Metastatic sites							
Liver metastasis	16 (47.1)	14	0.81 (0.45–1.87)	0.807	16	1.28 (0.62–2.64)	0.502
Lung metastasis	5 (14.7)	5	1.96 (0.74–5.20)	0.179	5	5.95 (1.92–18.5)	0.002
Peritoneal seeding	6 (17.7)	6	3.07 (1.21–7.82)	0.018	6	2.53 (0.99–6.49)	0.054
Lymph node metastasis	20 (58.8)	18	0.97 (0.47–1.99)	0.929	17	0.80 (0.39–1.64)	0.538
Number of metastatic sites		31	1.73 (1.04–2.86)	0.035	31	3.01 (1.59–5.68)	<0.001
1	19 (55.9)	17			16		
2	12 (35.3)	11			12		
3 or more	3 (8.8)	3			3		
IHC expression							
hENT1	25 (73.5)	23	1.95 (0.84–4.52)	0.119	23	1.73 (0.72–4.13)	0.219
DCK	11 (32.4)	11	1.00 (0.48–2.10)	0.997	11	1.16 (0.55–2.47)	0.697
CDA	12 (36.4)	12	1.39 (0.66–2.94)	0.390	11	1.00 (0.46–2.18)	0.998
RRM1	10 (30.3)	10	2.30 (1.03–5.14)	0.043	9	1.68 (0.75–3.77)	0.207

HR, hazard ratio; CI, confidence interval; hENT1, human Equilibrative Nucleoside Transporter; DCK, deoxycytidine kinase; CDA, deoxycytidine deaminase; RRM1, Ribonucleotide Reductase M1.

**Table 2:** Chemotherapy response according to each biomarker expression.

Variables	Chemotherapy Response		
	PR + SD (%)	PD (%)	*p* Value
hENT1			1
(−)	4 (44.4)	5 (55.6)	
(+)	10 (40)	15 (60)	
DCK			0.257
(−)	10 (43.5)	13 (56.5)	
(+)	4 (36.4)	7 (63.6)	
CDA			1
(−)	9 (42.9)	12 (57.1)	
(+)	5 (41.7)	7 (58.3)	
RRM1			0.257
(−)	8 (34.8)	15 (65.2)	
(+)	6 (60)	4 (40)	

PR, partial response; SD, stable disease; PD, progression disease; HR, hazard ratio; CI, confidence interval; hENT1, human Equilibrative Nucleoside Transporter; DCK, deoxycytidine kinase; CDA, deoxycytidine deaminase; RRM1, Ribonucleotide Reductase M1.

**Table 3:** Multivariate analysis for overall survival in RRM1 expression including peritoneal seeding or the number of metastatic sites.

	HR (95%CI)	*p* Value
Model 1 including peritoneal seeding		
RRM1 expression	3.33 (1.37–8.07)	0.008
Peritoneal seeding	4.99 (1.76–14.2)	0.003
Model 2 including number of metastatic sites		
RRM1 expression	3.44 (1.40–8.45)	0.007
Number of metastatic sites	2.30 (1.30–4.05)	0.004

## Data Availability

The datasets generated during and/or analyzed during the current study are available from the corresponding author on reasonable request.

## Supplementary Materials

The following are available online at https://www.mdpi.com/article/10.3390/jcm10204652/s1, Figure S1: Representative examples of negative immunohistochemical labeling profiles. (A) hENT1 in extrahepatic cholangiocarcinoma (eCCA) (B) DCK in gallbladder cancer, (C) CDA in eCCA, (D) Faint staining of RRM1 in eCCA; Table S1: Baseline characteristics according to each biomarker expression; Table S2: Multivariate analysis for progression free survival in each biomarker including peritoneal seeding and lung metastasis or the number of metastatic sites; Table S3: Multivariate analysis for overall survival in each biomarker including peritoneal seeding or number of metastatic sites.
