# Supplementary material for: RRM1 Expression as a Prognostic Biomarker for Unresectable or Recurrent Biliary Tract Cancer Treated with Gemcitabine plus Cisplatin
Source: J Clin Med. 2021 Oct 11;10(20):4652. doi: 10.3390/jcm10204652 (PMC8538709; doi:10.3390/jcm10204652)

Table S1. Baseline characteristics according to each biomarker expression

	hENT1		P value	RRM1		P value	DCK		P value	CDA		P value
	Negative	Positive		Negative	Positive		Negative	Positive		Negative	Positive	
Age (mean±SD)	60.3±5.4	66.2±8.9	0.072	64.7±9.5	65.1±6.5	0.876	65.3±9.2	63.4±6.9	0.54	64.7±9.5	63.4±6.9	0.893
Sex			0.704			0.461			0.135			1
Male	6	14		15	5		16	4		13	7	
Female	3	11		8	5		7	7		8	5	
Primary tumor site			0.024			0.201			0.255			0.271
Extrahepatic	3	5		4	4		4	4		3	5	
Gallbladder	1	15		11	5		13	3		11	4	
Intrahepatic	5	5		8	1		6	4		7	3	
Pathological differentiation			0.716			0.396			0.425			1
Well	1	1		2	0		1	1		1	1	
Moderately	5	11		9	7		9	7		11	5	
Poorly	3	10		9	3		10	3		8	4	
Stage									0.045			0.69
Metastatic disease							19	5		16	8	
Recurrent disease	4	6	0.395	5	5	0.215	4	6		5	4	
Metastatic sites												
Liver metastasis	3	13	0.448	11	5	1	12	4	0.477	10	6	1
Lung metastasis	2	3	0.591	3	2	0.627	2	3	0.3	3	2	1
Peritoneal seeding	1	5	1	5	1	0.64	3	3	0.363	4	2	1
Lymph node metastasis	5	15	1	13	6	1	16	4	0.135	11	8	0.486
Number of metastatic sites			0.014			0.73			0.646			0.867
1	7	12		12	6		12	7		12	6	
2	0	12		8	4		8	4		7	5	
3 or more	2	1		3	0		3	0		2	1	
IHC expression positive												
hENT1	1	9	0.217	15	9	0.217	18	7	0.425	13	11	0.107
DCK	4	7	0.425	6	5	0.24	5	5	0.24	7	2	0.422
CDA	1	11	0.107	10	2	0.422	9	3	0.71	7	3	0.71

hENT1, human Equilibrative Nucleoside Transporter; RRM1, Ribonucleotide Reductase M1; DCK, deoxycytidine kinase; CDA, deoxycytidine deaminase

Table S2. Multivariate analysis for progression free survival in each biomarker including peritoneal seeding and lung metastasis or number of metastatic sites

	hENT1		DCK		CDA		RRM1	
	HR (95%CI)	P value	HR (95%CI)	P value	HR (95%CI)	P value	HR (95%CI)	P value
**Including metastasis site**								
Biomarker	1.71<br>(0.70-4.17)	0.239	0.77<br>(0.34-1.71)	0.512	0.91<br>(0.42-2.01)	0.821	2.23<br>(0.93-5.37)	0.074
Peritoneal seeding	3.16<br>(1.15-8.70)	0.026	3.86<br>(1.38-10.8)	0.010	3.39<br>(1.24-9.28)	0.018	4.42<br>(1.52-12.9)	0.006
Lung metastasis	8.85<br>(2.64-29.6)	<.001	9.28<br>(2.67-32.3)	<.001	7.83<br>(2.37-25.8)	0.001	8.20<br>(2.44-27.5)	0.001
**Including number of metastatic sites**								
Biomarker	1.16<br>(0.46-2.91)	0.753	1.52<br>(0.69-3.35)	0.299	0.93<br>(0.42-2.07)	0.867	2.22<br>(0.96-5.16)	0.063
Number of metastatic sties	2.91<br>(1.49-5.68)	0.002	3.27<br>(1.68-6.38)	<.001	2.97<br>(1.55-5.67)	0.001	3.33<br>(1.69-6.58)	0.001

Table S3. Multivariate analysis for overall survival in each biomarker including peritoneal seeding or number of metastatic sites

	hENT1		DCK		CDA		RRM1	
	HR (95%CI)	P value	HR (95%CI)	P value	HR (95%CI)	P value	HR (95%CI)	P value
**Including metastasis site**								
Biomarker	1.74 (0.73–4.14)	0.210	0.83 (0.38–1.81)	0.647	1.38 (0.65–2.92)	0.407	3.33 (1.37–8.07)	0.008
Peritoneal seeding	2.69 (1.05–6.94)	0.040	3.26 (1.24–8.62)	0.017	3.07 (1.19–7.88)	0.020	4.99 (1.76–14.2)	0.003
**Including number of metastatic sites**								
Biomarker	1.70 (0.72–4.03)	0.228	1.21 (0.55–2.62)	0.638	1.23 (0.57–2.65)	0.591	3.44 (1.40–8.45)	0.007
Number of metastatic sties	1.62 (0.95–2.74)	0.074	1.79 (1.05–3.05)	0.032	1.69 (1.00–2.84)	0.049	2.30 (1.30–4.05)	0.004

Figure S1. Representative examples of negative immunohistochemical labeling profiles. (A) hENT1 in extrahepatic cholangiocarcinoma (eCCA) (B) DCK in gallbladder cancer, (C) CDA in eCCA, (D) Faint staining of RRM1 in eCCA.

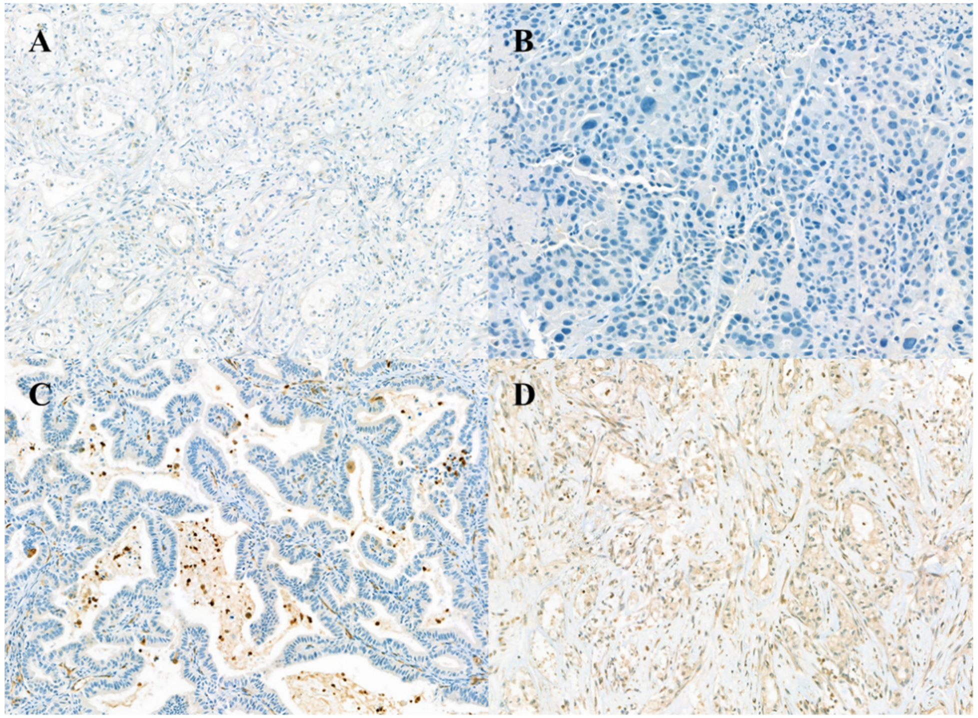

Supplement: Supplementary file 1 [file jcm-10-04652-s001.zip › jcm-1385254-SI.pdf]
